# Association between 90^o^ push-up and cardiorespiratory fitness: cross-sectional evidence of push-up as a tractable tool for physical fitness surveillance in youth

**DOI:** 10.1186/s12887-019-1840-9

**Published:** 2019-11-25

**Authors:** Toyin Ajisafe

**Affiliations:** Department of Kinesiology, Texas A&M University, Corpus Christi, TX USA

**Keywords:** Musculoskeletal fitness, Cardiorespiratory fitness, Physical fitness surveillance, Physical activity, Overweight and obesity

## Abstract

**Background:**

Despite being associated with health outcomes like abdominal adiposity, depression, anxiety, and cardiovascular disease risk among youth, largely, clinicians still do not adopt physical fitness testing. A clarion call for increased surveillance was previously issued, in order to address the US population-level lack of knowledge regarding pervasive inactivity among children. Because schools often do not send home annual physical fitness testing results, many lay parents are unaware of their child’s physical fitness or the risk of associated adverse health outcomes. This study investigated associations between musculoskeletal fitness measures (including 90^o^ push-up), cardiorespiratory fitness, and weight status.

**Methods:**

Two hundred and ten students (9.7 ± 1.08 years, 138.6 ± 9.4 cm; 42.3 ± 14.4 kg) across third through fifth grades were tested for cardiorespiratory (i.e., Progressive Aerobic Cardiovascular Endurance Run (PACER)) and musculoskeletal (90^o^ push-up, trunk lift, sit-and-reach and curl-up) fitness. The relationships between measures of musculoskeletal and cardiorespiratory fitness were modeled using a series of linear regression analyses. Models were adjusted for age, sex, and weight status. Significant two-tailed tests were set at *p < .05*.

**Results:**

Of the four musculoskeletal fitness measures, only 90^o^ push-up was significantly associated (β = .353; *p < .001*) with PACER test scores (i.e., cardiorespiratory fitness). The related model (R^2^ = .324; F (4,205) = 26.061; *p* < .001) accounted for 32% of the variance in cardiorespiratory fitness. 90^o^ push-up was associated with sit-and reach (β = .298; *p < .001*) and curl up (β = .413; *p < = .001*) test scores. When individually modeled, 90^o^ push-up (β = −.461; *p < .001*) and PACER (β = −.436; *p < .001*) were inversely associated with weight status.

**Conclusions:**

The 90^o^ push-up test (a measure of upper body muscle strength and endurance) was associated with cardiorespiratory fitness, anterior trunk muscle strength and endurance, and lower back and posterior thigh muscle flexibility in youth aged 8–12 years old. Although the current findings do not establish a causal relationship, it is concluded that the 90^o^ push-up test is a tractable tool for physical fitness surveillance by clinicians, parents, and possibly youth themselves.

## Background

Cardiorespiratory fitness or endurance is the capacity to execute whole-body movements like running and jumping, which often involve large muscle groups, at a moderate to vigorous intensity for relatively sustained durations [1]. Cardiorespiratory fitness also impacts the ability to perform less vigorous tasks like negotiating stairs, performing household chores, and walking briskly [1]. Therefore, having adequate cardiorespiratory fitness allows individuals to perform these whole-body tasks without experiencing quick onset and debilitating or disruptive levels of fatigue [2]. The cardiorespiratory system (i.e., the heart, lungs, and blood vessels, including the blood that they carry) is critical to improving cardiorespiratory fitness. While skeletal muscle can undergo physiological adaptations that can optimize its capacity to utilize oxygen (i.e., aerobic metabolism), mechanistically, the cardiorespiratory system has to effectively transport oxygen to active skeletal muscles where it is metabolized [2]. This underscores the inter-dependency between the musculoskeletal and cardiorespiratory systems.

Musculoskeletal and cardiorespiratory fitness have been linked with a number of health outcomes, including whole body and abdominal adiposity, depression, anxiety, self-esteem and cardiovascular disease risk, in youth [3]. Changes in cardiorespiratory fitness and muscular strength accounted for 15% of the variance in pre-and adolescent’s adiposity and abdominal adiposity across 5 years [4]. Lower musculoskeletal fitness was associated with unhealthy body mass index (BMI) and poorer health outcomes in children [3, 5]. We previously reported pervasive low musculoskeletal fitness scores in a predominantly Latino sample of school children in Corpus Christi, Texas [6]. Obesity was 32% prevalent in the same sample. The inverse association between cardiorespiratory fitness and BMI is well documented [3, 5, 7–9]. Children with greater cardiorespiratory fitness and low fatness had increased odds of superior academic achievement [10]. This finding persisted when children had high fatness but better cardiorespiratory fitness and muscle strength, thereby leading the authors to conclude that both parameters moderated the adverse association between body fatness and academic achievement [10]. Although there is growing evidence to support the objective assessment of cardiorespiratory fitness as a vital sign in health care settings [11], clinicians still predominantly use patients’ self-reports rather than objectively measured physical activity and cardiorespiratory fitness [12]. Unfortunately, these self-reports significantly overestimate physical activity and fitness [12]. Additionally, tests of cardiorespiratory fitness can be time consuming, space-prohibitive, and require specialized equipment. These barriers may account for their continued lack of adoption in clinical settings [12].

Disparities in physical activity, obesity, and type 2 diabetes exist among Latino youth. Hispanic children are more affected by overweight and obesity [13] and up to 50% of Latino children are projected to develop type 2 diabetes in their lifetime [14]. Hispanic-American children were less active at home and during recess at school than non-Hispanic White-American peers [15–18]. Physical Activity Guidelines for Americans recommends at least 60 daily minutes of moderate-to-vigorous physical activity for children and adolescents aged 6–17 years [19]. Although the level of physical activity necessary to maintain a healthy weight or decrease excess body weight will expectedly vary between individuals, physical activity is thought to prevent weight gain when done at moderate- or vigorous-intensity and is aerobic in nature [19, 20]. Despite many efforts to minimize sedentary behavior, there remains a lack of awareness amongst parents regarding the gravity and degree to which many children in the US are inactive [21]. Considering the positive associations between cardiorespiratory and musculoskeletal fitness and sustained physical activity engagement [21], it is critical that youth can engage in sustained physical activity without experiencing disruptions owing to quick onset fatigue. Importantly, it is imperative that parents, clinicians, and youth themselves and have a simplistic proxy surveillance mechanism to evaluate their current physical fitness and risk of adverse cardiometabolic outcomes.

FitnessGram® testing is widely used to evaluate children’s health-related fitness [22]. Specifically, it assesses cardiorespiratory fitness using the Progressive Aerobic Cardiovascular Endurance Run (PACER) and musculoskeletal fitness using the 90^o^ push-up, trunk lift, and curl-up tests [23]. While trunk lift test had the highest pass rate, push-up and curl-up tests had the lowest pass rates among school-aged Portuguese children [24]. It is unclear whether these measures have varied associations with cardiorespiratory fitness. When schools adopt it, FitnessGram® testing is typically performed once a year, and some schools do not perform it all in certain years. When performed, FitnessGram® testing results are often not sent home to parents. Consequently, many lay parents likely have no insight into the fitness level of their child. This is consistent with the fact that there is a pervasive lack of knowledge on the degree of inactivity among children and youth in the US [21]. In fact, a clarion call was recently issued regarding the need for cardiorespiratory fitness surveillance among youth in the US as a means to help facilitate risk classification, monitor health status changes, and inform recommendations for lifestyle changes by clinicians [21, 25]. Of four measures (90^o^ push-up, curl-up, trunk lift, sit-and-reach) of musculoskeletal fitness (i.e., muscle strength, endurance, and flexibility, the 90^o^ push-up test was most consistently discriminatory of being obese relative to having a healthy weight across all the elementary grades tested [6]. Push-up was recently found to be associated with cardiovascular events in active adult men [12].

To our knowledge, no study has investigated the 90^o^ push-up test as a potential surrogate measure by examining its relationship with cardiorespiratory and other measures of musculoskeletal fitness in youth. Findings could help make the case for 90^o^ push-up as a valuable proxy that clinicians (particularly in pediatric settings) and parents can administer (at home), in order to surveil physical fitness and potential risk of adverse health outcomes related to inadequate physical activity among youth. Therefore, this research aimed to establish the associations between measures of musculoskeletal fitness (including 90^o^ push-up), cardiorespiratory fitness, and weight status while adjusting for age, sex, and weight status in a sample of predominantly Latino youth.

It was hypothesized that the 90^o^ push-up test (of four musculoskeletal fitness measures) will be most strongly associated with cardiorespiratory fitness. 90^o^ push-up will also be associated with other measures of musculoskeletal fitness and weight status.

## Methods

### Participants

The data was from a larger cross sectional sample of 492 elementary school youth in Corpus Christi, Texas, and previously described by T Ajisafe, T Garcia and H Fanchiang [6]. There sample was 84.3% Latino, 7% African American, 6.7% White. Ninety three percent of the student population is listed as economically disadvantaged. This study is a post hoc subgroup analyses of 253 students who met the eligibility criteria from the larger cross-sectional sample, i.e., students had to be in third through fifth grades as these were the only ones who participated in the PACER tests. Students were excluded from further analyses, if they were missing any data at all. PACER test scores accounted for most (86%) of the missing data fields. Therefore, 16 students were excluded from further analysis due to missing data. Additionally, six students were excluded, because the they were underweight. This underweight count was considered too diminutive to be included in the analyses. There were 210 participants (9.7 ± 1.08 years; 138.6 ± 9.4 cm; 42.3 ± 14.4 kg). There were 116 males. A detailed list of anthropometrics (classified by weight status) is provided in Table 1. Texas A&M University-Corpus Christi Institutional Review Board approved this study (IRB # 122–17).

**Table 1:** Descriptive and anthropometric data (Mean (SD)) for youth with healthy weight, overweight, and obesity

	Healthy weight	Overweight	Obese
Number of Participants	86 (41%)	38 (18%)	86 (41%)
Male to Female ratio	48:38	18:20	50:36
Age (years)	9.7 ± 1.2	9.8 ± 1.2	9.6 ± 0.9
Height (cm)	134.9 ± 8.9	139.4 ± 9.9	142.0 ± 8.4
Body mass (kg)	30.5 ± 5.6	40.3 ± 8.6	55.1 ± 11.7
BMI (kg/m^2^)	16.6 ± 1.4	20.5 ± 2.0	27.1 ± 4.1
Obesity Classification			
Number of Obese Class 1			46 (54%)
Number of Obese Class 2			28 (33%)
Number of Obese Class 3			11 (12%)

### Procedures

Protocols and equipment for the trunk lift, 90^o^ push-up, curl-up, and the back saver sit and reach tests were described elsewhere [26]. These tests and the PACER were administered by the same resident physical education specialist at the school. The physical education specialist previously underwent ad hoc training and had administered FitnessGram® testing for several years in consistence with the Texas state mandate (Senate Bill 530) requiring yearly health-related fitness testing of school children. The original aim of the mandate was to track overweight and obesity and pre-disposition to chronic diseases like type 2 diabetes;

### Data analysis

Participants with any missing data were excluded: one data set was excluded in kindergarten. Height and weight data were converted from inches and pounds to meters and kilograms, respectively. BMI was computed as the quotient of weight (kg) and the square of height (m). These scores were standardized as z-scores and used to determine respective percentiles for age and sex according to the Centers for Disease Control and Prevention (CDC) growth charts [27]. Underweight, healthy weight, overweight, and obesity were defined as BMI < 5th percentile, 5th ≤ BMI < 85th percentile, 85th ≤ BMI < 95th percentiles, and BMI ≥ 95th percentile, respectively [27, 28]. Obesity was further delineated as class 1 (95th ≤ BMI < 120% of the 95th percentile), class 2 (120% of the 95th percentile ≤ BMI < 140% of the 95th percentile), and class 3 (BMI ≥ 140% of the 95th percentile or BMI ≥ 40.0 kgm^− 2^). Given the unequal distances between the percentile-based classifications, the weight classes were treated as categorical data: healthy weight was coded as “1,” overweight was coded as “2,” and obesity was coded as “3.” Musculoskeletal fitness, i.e., measures of muscle strength, endurance, and flexibility, were assessed by the school’s trained resident physical education specialist. Scores on trunk lift and the back saver sit and reach tests were measured in inches, while push-up and curl-up were simply the number or repetitions completed.

### Statistical analysis

Data was explored for normality using the Kolmogorov-Smirnov test. Data were explored for outliers using box plots. Variance Inflation Factors were computed and examined to detect any instances of multicollinearity. Pearson and Spearman correlations were used to examine bivariate correlations between measures of cardiorespiratory and musculoskeletal fitness, and age, sex and weight status. The relationships between measures of musculoskeletal and cardiorespiratory fitness were modeled using a series of linear regression analyses. Sex was dummy-coded prior to entering it to the regression models. The magnitudes of the respective associations (represented as standardized beta coefficients) and the corresponding 95% confidence intervals (CIs) are presented in the results section. Multiple models that were unadjusted and adjusted for age, sex, and weight status were explored. Assumptions for linear regression models were verified. Significant two-tailed tests were set at 5% (i.e., *p < .05*).

## Results

### Musculoskeletal and cardiorespiratory fitness test scores

**Fig. 1:**
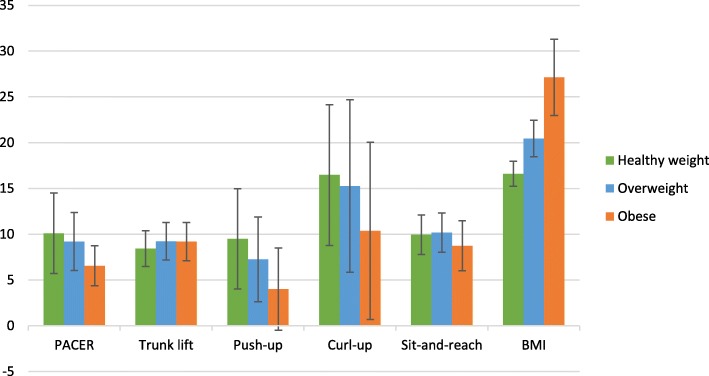
Mean (SD) musculoskeletal and cardiorespiratory fitness and BMI scores in 3rd grade, (e) 4th grade, and (f) 5th grade. Scores on trunk lift and the back saver sit and reach tests were measured in inches, push-up and curl-up are the number of repetitions completed, and raw BMI was calculated as kgm^− 3^

Mean (SD) musculoskeletal and cardiorespiratory fitness test scores are presented in Fig. 1. Additionally, raw BMI scores are shown (Fig. 1).

### Associations between musculoskeletal fitness and cardiorespiratory fitness

Bivariate correlations between measures of musculoskeletal and cardiorespiratory fitness, age, sex, and weight status are presented in Table 2.

**Table 2:** Bivariate correlations between measures of musculoskeletal and cardiorespiratory fitness, age, sex, and weight status

	Age	Female	Weight status	PACER	Trunk lift	90^o^ Push-up	Curl-up	Sit-and-reach
Age	1	−.134	−.034	.159*	.072	.085	.220**	−.162*
*p*-value		.052	.622	.021	.297	.222	.001	.019
Sex	−.134	1	−.021	−.084	.223**	−.082	.098	.287**
*p*-value	.052		.760	.226	.001	.236	.156	<.001
Weight status	−.034	−.021	1	−.426**	.169*	−.450**	−.298**	−.221**
*p*-value	.622	.760		<.001	.014	<.001	<.001	.001
PACER	.159*	−.094	−.426**	1	−.070	.527**	.347**	.181**
*p*-value	.021	.173	<.001		.313	<.001	<.001	.009
Trunk lift	.072	.215**	.169*	−.070	1	−.057	.099	.193**
*p*-value	.297	.002	.014	.313		.413	.154	.005
90^o^ Push-up	.085	−.131	−.450**	.527**	−.057	1	.463**	.301**
*p*-value	.222	.058	<.001	<.001	.413		<.001	<.001
Curl-up	.220*	.102	−.298**	.347**	.099	.463**	1	.203**
*p*-value	.001	.141	<.001	<.001	.154	<.001		.003
Sit-and-reach	.162*	.286**	−.221**	.181**	.193**	.301**	.203**	1
*p*-value	.019	<.001	.001	.009	.005	<.001	.003	

Correlation coefficients between all continuous variables are Pearson’s r, and those involving weight status are Spearman’s rho. * Indicates statistical significance at the level of *p* < .05. ** Indicates statistical significance at the level of *p* < .01

### Associations between musculoskeletal and cardiorespiratory fitness measures

An unadjusted model (R^2^ = .282; F (4,205) = 21.494, *p* < .001) that included trunk lift (β = −.064; *p* = .296), 90^o^ push-up (β = .451; *p* < .001), curl-up (β = .139; *p* = .039), and sit-and-reach (β = .029; *p* = .647) accounted for 28% of the variance in cardiorespiratory fitness. A second unadjusted model (R^2^ = .275; F (1,208) = 80.105; *p* < .001) that only included 90^o^ push-up (β = .527; *p* < .001) equally accounted for 28% of the variance in cardiorespiratory fitness (Table 3).

**Table 3:** Age-, sex-, and weight status-unadjusted models for the associations between measures of musculoskeletal and cardiorespiratory fitness (dependent variable: PACER test score)

Model	Predictor variable	VIF	*p* - value	β	95% CI
Model 1	Trunk lift	1.065	.296	−.064	(−.336, .103)
	90^o^ push-up**	1.389	<.001	.451	(.215, .402)
	Curl-up*	1.331	.039	.139	(.003, .110)
	Sit-and-reach	1.136	.647	.029	(−.143, .233)
Model 2	90^o^ push-up**	1.000	<.001	.527	(.281, .440)

* Indicates statistical significance at the level of *p* < .05. ** Indicates statistical significance at the level of *p* < .01. *β* Standardized Beta Coefficient, *VIF* Variance Inflation Factor

When adjusted for age, sex, and weight status, a model (R^2^ = .322; F (7,202) = 15.192, *p* < .001) that included trunk lift (β = −.025; *p* = .680), 90^o^ push-up (β = .353; *p* < .001), curl-up (β = .094; *p* = .169), and sit-and-reach (β = .043; *p* = .513) accounted for 32% of the variance in cardiorespiratory fitness (Table 4). A second adjusted model (R^2^ = .324; F (4,205) = 26.061; *p* < .001) that only included 90^o^ push-up (β = .405; *p* < .001) equally accounted for 32% of the variance in cardiorespiratory fitness (Table 4).

**Table 4:** Age-, sex-, and weight status-adjusted models for the associations between measures of musculoskeletal and cardiorespiratory fitness (dependent variable: PACER test score)

Model	Predictor variable	VIF	*p* - value	β	95% CI
Model 1	Trunk lift	1.147	.680	−.025	(−.267, .175)
	90^o^ push-up**	1.626	<.001	.353	(.142, .341)
	Curl-up	1.449	.169	.094	(−.016, .093)
	Sit-and-reach	1.293	.513	.043	(−.132, .264)
	Age	1.163	.093	.102	(−.060, .775)
	Female	1.197	.381	−.056	(−1.364, .523)
	Weight status**	1.301	.001	−.223	(−1.470, −.387)
Model 2	90^o^ push-up**	1.258	<.001	.405	(.190, .364)
	Age	1.036	.052	.112	(−.004, .789)
	Female	1.033	.592	−.031	(−1.103, .631)
	Weight status**	1.229	<.001	−.241	(− 1.525, −.476)

** Indicates statistical significance at the level of *p* < .01. *β* Standardized Beta Coefficient, *VIF* Variance Inflation Factor

### Associations between measures of musculoskeletal fitness

An unadjusted model (R^2^ = .054; F (3,206) = 4.957, *p* = .002) that included 90^o^ push-up (β = −.189; *p* = .017), curl-up (β = .141; *p* = .065), and sit-and-reach (β = .221; *p* = .002) accounted for 5% of the variance in trunk lift scores (Table 5). When adjusted for age, sex, and weight status, a model (R^2^ = .111; F (6,203) = 5.328, *p* < .001) that included 90^o^ push-up (β = −.057; *p* = .497), curl-up (β = .109; *p* = .162), and sit-and-reach (β = .213; *p* = .004) accounted for 11% of the variance in trunk lift scores (Table 6).

**Table 5:** Age-, sex-, and weight status-unadjusted model for the associations between measures of musculoskeletal fitness (dependent variable: trunk lift)

Model	Predictor variable	VIF	*p* - value	β	95% CI
Model 1	90^o^ push-up*	1.360	.017	−.189	(−.128, −.013)
	Curl-up	1.309	.065	.141	(−.002, .065)
	Sit-and-reach**	1.091	.002	.221	(.068, .299)

* Indicates statistical significance at the level of *p* < .05. ** Indicates statistical significance at the level of *p* < .01. *β* Standardized Beta Coefficient, *VIF* Variance Inflation Factor

**Table 6:** Age-, sex-, and weight status-adjusted model for the associations between measures of musculoskeletal fitness (dependent variable: trunk lift)

Model	Predictor variable	*VIF*	*p* - value	β	95% CI
Model 2	90^o^ push-up	1.632	.497	−.057	(−.083, .041)
	Curl-up	1.426	.162	.109	(−.010, .058)
	Sit-and-reach**	1.253	.004	.213	(.056, .298)
	Age	1.146	.092	.117	(−.036, .482)
	female*	1.169	.030	.156	(.062, 1.230)
	Weight status**	1.246	.002	.230	(.191, .854)

* Indicates statistical significance at the level of *p* < .05. ** Indicates statistical significance at the level of *p* < .01. *β* Standardized Beta Coefficient, *VIF* Variance Inflation Factor

An unadjusted model (R^2^ = .269; F (3,206) = 26.602, *p* < .001) that included trunk lift (β = −.146; *p* = .017), curl-up (β = .428; *p* < .001), and sit-and-reach (β = .242; *p* < .001) accounted for 27% of the variance in 90^o^ push-up scores (Table 7). When adjusted for age, sex, and weight status, a model (R^2^ = .382; F (6,203) = 22.529, *p* < .001) that included trunk lift (β = −.040; *p* = .497), curl-up (β = .353; *p* < .001), and sit-and-reach (β = .241; *p* < .001) accounted for 38% of the variance in 90^o^ push-up scores (Table 8).

**Table 7:** Age-, sex-, and weight status-unadjusted model for the associations between measures of musculoskeletal fitness (dependent variable: 90^o^ push-up)

Model	Predictor variable	*VIF*	*p* - value	β	95% CI
Model 1	Trunk lift*	1.043	.017	.146	(−.709, −.071)
	Curl-up**	1.049	<.001	.428	(.184, .326)
	Sit-and-reach**	1.074	<.001	.242	(.269, .807)

*Indicates statistical significance at the level of *p* < .05. ** Indicates statistical significance at the level of *p* < .01. *β* Standardized Beta Coefficient, *VIF* Variance Inflation Factor

**Table 8:** Age-, sex-, and weight status-adjusted model for the associations between measures of musculoskeletal fitness (dependent variable: 90^o^ push-up)

Model	Predictor variable	VIF	*p* - value	β	95% CI
Model 2	Trunk lift	1.140	.497	−.040	(−.415, .202)
	Curl-up**	1.205	<.001	.353	(.140, .281)
	Sit-and-reach**	1.222	<.001	.241	(.268, .801)
	Age	1.153	.899	.007	(−.545, .620)
	female**	1.126	<.001	−.232	(− 3.839, − 1.303)
	Weight status**	1.186	<.001	−.290	(− 2.477, − 1.046)

** Indicates statistical significance at the level of *p* < .01. *β* Standardized Beta Coefficient, *VIF* Variance Inflation Factor

An unadjusted model (R^2^ = .220; F (3,206) = 20.679, *p* < .001) that included trunk lift (β = .116; *p* = .065), 90^o^ push-up (β = .456; *p* < .001), and sit-and-reach (β = .043; *p* = .511) accounted for 22% of the variance in curl-up test scores (Table 9). When adjusted for age, sex, and weight status, a model (R^2^ = .277; F (6,203) = 14.364, *p* = .000) that included trunk lift (β = .088; *p* = .162), 90^o^ push-up (β = .413; *p* = .000), and sit-and-reach (β = .025; *p* = .712) accounted for 28% of the variance in curl-up test scores (Table 10).

**Table 9:** Age-, sex-, and weight status-unadjusted model for the associations between measures of musculoskeletal fitness (dependent variable: curl-up)

Model	Predictor variable	VIF	*p* - value	β	95% CI
Model 1	Trunk lift	1.046	.065	.116	(−.033, 1.078)
	90^o^ push-up**	1.093	<.001	.456	(.553, .980)
	Sit-and-reach	1.132	.511	.043	(−.322, .644)

** Indicates statistical significance at the level of *p* < .01. *β* Standardized Beta Coefficient, *VIF* Variance Inflation Factor

**Table 10:** Age-, sex-, and weight status-adjusted model for the associations between measures of musculoskeletal fitness (dependent variable: curl-up)

Model	Predictor variable	VIF	*p* - value	β	95% CI
Model 2	Trunk lift	1.122	.162	.088	(−.160, .955)
	90^o^ push-up**	1.357	<.001	.413	(.462, .926)
	Sit-and-reach	1.290	.712	.025	(−.408, .596)
	Age**	1.092	.001	.200	(.682, 2.745)
	female*	1.168	.017	.154	(.511, 5.229)
	Weight status	1.294	.102	−.111	(− 2.502, .226)

* Indicates statistical significance at the level of *p* < .05. ** Indicates statistical significance at the level of *p* < .01. *β* Standardized Beta Coefficient, *VIF* Variance Inflation Factor

An unadjusted model (R^2^ = .124; F (3,206) = 10.872, *p* < .001) that included trunk lift (β = .205; *p* = .002), 90^o^ push-up (β = .290; *p* < .001), and curl-up (β = .049; *p* = .511) accounted for 12% of the variance in sit-and-reach test scores (Table 11). When adjusted for age, sex, and weight status, a model (R^2^ = .235; F (6,203) = 11.691, *p* < .001) that included trunk lift (β = .183; *p* = .004), 90^o^ push-up (β = .298; *p* < .001), and curl-up (β = .027; *p* = .712) accounted for 24% of the variance in sit-and-reach test scores (Table 12).

**Table 11:** Age-, sex-, and weight status-unadjusted model for the associations between measures of musculoskeletal fitness (dependent variable: sit-and-reach)

Model	Predictor variable	VIF	*p* - value	β	95% CI
Model 1	Trunk lift**	1.023	.002	.205	(.091, .402)
	90^o^ push-up**	1.314	<.001	.290	(.065, .196)
	Curl-up	1.328	.511	.049	(−.026, .052)

** Indicates statistical significance at the level of *p* < .01. *β* Standardized Beta Coefficient, *VIF* Variance Inflation Factor

**Table 12:** Age-, sex-, and weight status-adjusted model for the associations between measures of musculoskeletal fitness (dependent variable: sit-and-reach)

Model	Predictor variable	VIF	*p* - value	β	95% CI
Model 2	Trunk lift**	1.109	.004	.183	(.069, .373)
	90^o^ push-up**	1.547	<.001	.298	(.067, .201)
	Curl-up	1.450	.712	.027	(−.031, .045)
	Age**	1.100	.006	−.176	(−.690, −.117)
	female**	1.128	<.001	.257	(.644, 1.915)
	Weight status	1.279	.115	−.110	(−.679, −.074)

** Indicates statistical significance at the level of *p* < .01. *β* Standardized Beta Coefficient, *VIF* Variance Inflation Factor

### Association between musculoskeletal and cardiorespiratory fitness measures and weight status

A model adjusted for age and sex (R^2^ = .272; F (7,202) = 12.160, *p* < .001) that included trunk lift (β = .181; *p* = .004), 90^o^ push-up (β = −.255; *p* = .001), and PACER (β = −.240; *p* = .001) test scores as statistically significant contributors accounted for 27% of the variance in weight status (Table 13). A second age- and sex-adjusted model (R^2^ = .245; F (6,203) = 12.319, *p* < .001) with 90^o^ push-up (β = −.274; *p* = .001), and PACER (β = −.256; *p* < .001) test scores as statistically significant contributors accounted for 25% of the variance in weight status (Table 13). A third age- and sex-adjusted model (R^2^ = .198; F (3,206) = 18.184, *p* < .001) with only 90^o^ push-up (β = −.461; *p* < .001) test scores as the statistically significant contributor accounted for 20% of the variance in weight status (Table 13). A fourth model adjusted for age and sex (R^2^ = .174; F (3,206) = 15.713, *p* < .001) with only PACER (β = −.436; *p* < .001) test scores as the statistically significant contributor accounted for 17% of the variance in weight status (Table 13). A fifth model (R^2^ = .021; F (3,206) = 2.502, *p* = .060) adjusted for age and sex with trunk lift (β = .188; *p* = .008) as the lone musculoskeletal fitness measure was not statistically significant.

**Table 13:** Age- and sex-adjusted models for associations between measures of musculoskeletal and cardiorespiratory fitness and weight status (dependent variable: weight status)

Model	Predictor variable	VIF	*p* - value	β	95% CI
Model 1	Trunk lift**	1.095	.004	.181	(.026, .134)
	90^o^ push-up**	1.837	.001	−.255	(−.067, −.017)
	Curl-up	1.447	.207	−.089	(−.022, .005)
	Sit-and-reach	1.270	.169	−.094	(−.084,.015)
	PACER**	1.510	.001	−.240	(−.091, −.024)
	Age	1.189	.925	.006	(−.100, .110)
	female	1.199	.227	−.079	(−.090, .379)
Model 2	90^o^ push-up**	1.810	.001	−.274	(−.071, −.019)
	Curl-up	1.434	.316	−.072	(−.021, .007)
	Sit-and-reach	1.242	.400	−.058	(−.070,.028)
	PACER**	1.514	<.001	−.256	(−.096, −.027)
	Age	1.186	.652	.029	(−.082, .130)
	female	1.179	.418	−.054	(−.334, .139)
Model 3	90^o^ push-up**	1.031	<.001	−.461	(−.096, −.055)
	Age	1.029	.919	−.006	(−.109, .098)
	female	1.032	.193	−.082	(−.376, .076)
Model 4	PACER**	1.058	<.001	−.436	(−.135, −.075)
	Age	1.062	.672	.027	(−.083, .129)
	female	1.031	.358	−.059	(−.335, .122)
Model 5	Trunk lift	1.049	.008	.188	(.022, .144)
	Age	1.025	.411	−.057	(−.163, .067)
	female	1.071	.329	−.069	(−.381, .128)

** Indicates statistical significance at the level of *p* < .01. *β* Standardized Beta Coefficient, *VIF* Variance Inflation Factor

## Discussion

This study primarily investigated the associations between measures of musculoskeletal fitness and cardiorespiratory fitness among youth aged 8–12 years. Of the measures of musculoskeletal fitness, only 90^o^ push-up was positively associated with cardiorespiratory fitness. Trunk lift, curl-up, and sit-and-reach were not significantly associated with cardiorespiratory fitness. The current study also explored associations between individual measures of musculoskeletal fitness. After adjusting for age, sex, and weight status, 90^o^ push-up was positively associated with curl-up and sit-and-reach, but not trunk lift scores. Trunk lift was only associated (positively) with sit-and-reach, and curl-up was only associated with 90^o^ push-up. Sit-and-reach was positively associated with trunk lift and 90^o^ push-up scores.

As hypothesized, 90^o^ push-up test was most strongly associated with cardiorespiratory fitness. In fact, it was the only musculoskeletal fitness measure that was associated with cardiorespiratory fitness. Although 90^o^ push-up test is often considered a test of upper body muscle strength and endurance, it engages both trunk and lower extremity muscles. These muscles (primarily trunk and lower extremity flexors and extensors) contract isometrically to help maintain the length of the body as a unitary lever during the downward and upward phases of its rotation about the axis of rotation at the toes. As such, the muscles involved in executing the 90^o^ push-up nearly span the whole body. Compared to trunk lift and curl-up, 90^o^ push-up also relies on upper extremity muscles with comparatively smaller physiologic cross-sectional areas to perform positive and negative work during the respective phases of the whole-body lever rotation. The implications of these factors may be such that the 90^o^ push-up is more intense and aerobically demanding. Previously, trunk lift test had the highest pass rate, while push-up and curl-up tests had the lowest pass rates among school-aged children [24]. Considering that cardiorespiratory fitness is a function of the body’s capacity to support skeletal muscle activity during intense aerobic metabolism, its lone association with 90^o^ push-up test scores makes logical sense.

While no existing studies have specifically explored the association between push-up capacity and cardiometabolic outcomes in youth, J Yang, CA Christophi, A Farioli, DM Baur, S Moffatt, TW Zollinger and SN Kales [12] recently found that push-up capacity was longitudinally associated with the incidence of cardiovascular events among active adult men. Consequently, they stressed the surveillance value, low-cost, and ease of adopting a push-up capacity examination in clinical settings [12]. An age- and sex-adjusted model with only 90^o^ push-up test scores (of other physical fitness measures) accounted for the most variance (i.e., 20%) in weight status. Previously, 90^o^ push-up was most consistently discriminatory (compared to curl-up, trunk lift, and sit and reach tests) of being obese relative to having a healthy weight in children [6]. Pertinent to the current study, the odds of being obese as compared to having healthy weight decreased by 17% for every unit increase in push-ups performed by students in third through fifth grades [6]. Findings from this current study support a similar argument regarding empowering parents, clinicians, and youth themselves (if old enough to self-monitor) to assess their physical fitness. In the context home surveillance, this argument is further strengthened by the fact that administering a 90^o^ push-up test does not require ample space, time, training, or equipment other than a simple metronome, which is ubiquitous in the form of several free applications on mobile devices, including cell phones. Additionally, age-specific references already exist, but new ones could certainly be explored. For example, FitnessGram® standards specify that girls aged 5–6 years, 7 years, 8 years, 9 years, and 10–11 years must perform 3–8, 4–10, 5–13, 6–15, and 7–15 repetitions, respectively, in order to demonstrate a healthy level of fitness (i.e., Healthy Fitness Zone) on the 90^o^ push-up test. Boys aged 5–6 years, 7 years, 8 years, 9 years, 10 years, 11 years, and 12 years must perform 3–8, 4–10, 5–13, 6–15, 7–20, 8–20, and 10–20 repetitions, respectively, in order to demonstrate a healthy level of fitness (i.e., Healthy Fitness Zone) on the 90^o^ push-up test [26]. These recommendations span up to age 17 years and older [29].

Amongst the four measures of musculoskeletal fitness (i.e., muscle strength, endurance, and flexibility), only the 90^o^ push-up and back saver sit-and-reach tests were associated with two other musculoskeletal measures. Specifically, 90^o^ push-up was positively associated with curl-up and sit-and-reach, but not trunk lift scores; sit-and-reach was positively associated with trunk lift and 90^o^ push-up scores, but not curl-up. As previously articulated, FitnessGram® testing results are often not sent home to parents, and there are years when some schools do not perform these tests at all. The resulting lack of awareness on the part of parents regarding their children’s fitness and indirect implications for potential risk of adverse health outcomes may partly underlie parents’ poor recognition of high inactivity levels among US youth [21]. Given its association with physical and mental health outcomes [3], there has been a call for regular cardiorespiratory fitness surveillance among youth in the US [21]. This call projected that issues like the current population-level decline in military readiness and the national security implications in the US may reach critical mass and drive national policy on mandatory cardiorespiratory fitness assessment. However, the authors conceded the difficulty of achieving the mobilization necessary to engender such policy change [21]. While it is unclear whether this would happen, the physical and mental health implications of poor fitness is one that likely resonates with most parents. It may be time to include parents in the surveillance conversation by empowering them with tractable tools to assess their child’s physical fitness and potential risk of related health problems without the need for specialized equipment or extensive training and time. Based the current associations, it is concluded that 90^o^ push-up test could potentially serve as a proxy for musculoskeletal and cardiorespiratory fitness in the hands of parents, clinicians, and youth themselves.

### Strengths and weaknesses

The main strengths of the current study include the fact that all tests and anthropometric measurements were administered by the same trained resident physical education specialist at the school site. Therefore, interrater variability was not a factor. The physical education specialist had several years of experience administering FitnessGram® tests. All the analyzed data (i.e., anthropometric, cardiorespiratory, and musculoskeletal) was objectively acquired. The issues that are commonly associated with self-reports, including over-and under-estimation were likely moderated. The sample was predominantly Latino, thereby giving representation to a group of children and region (in South Texas) that are often underrepresented in research, yet faced with disparities (i.e., increased incidents) related to physical inactivity, obesity, and type 2 diabetes.

This study has several limitations. The sample is from a single setting and the size was relatively small; however, it exceeded the size indicated in a priori power analysis (β = 0.95) involving seven tested predictors) for a medium effect size (i.e., *f*^*2*^ = 0.15). The cross-sectional design of this study does not provide any longitudinal insight into whether the observed associations persist beyond the age groups within this study. Further, current findings may not approximate the nature of the relationships between cardiorespiratory and musculoskeletal fitness measures among youth who are older than 12 years. The associations described in this study are not indicative of causal relationships between the variables. The age range within the sample was narrow, so findings may not generalize to youth younger than eight and older than 12 years. The research does not yield a prediction equation between 90^o^ push-up and PACER test scores; 90^o^ push-up test only accounted 32% of the variance in cardiorespiratory fitness measure when normalized for age, sex, and weight status. It is possible that some of the significant findings could be due to bias resulting from a lack of adjustment for multiple testing.

## Conclusions

The present findings suggest that 90^o^ push-up performance is positively associated with cardiorespiratory fitness, anterior trunk muscle strength and endurance, hamstring and lower back flexibility and inversely associated with weight status. Given these associations, 90^o^ push-up test seems a plausible and simple unitary proxy to assess physical fitness among youth in a variety of settings without requiring ample space, time, or any cost. Considering previous links between musculoskeletal and cardiorespiratory fitness and outcomes related to physical and mental health, training parents to assess their child’s current fitness using a unitary surrogate may will provide on demand insight without having to await yearly fitness assessment results that may never make it home from school. This is especially critical for families with low income who may be medically uninsured and never otherwise realize the incidence of poor physical fitness in their children. Further, rather than rely on self-reported physical activity, clinicians are encouraged to strongly consider adopting 90^o^ push-up test alongside extant vital signs, in order to objectively assess physical fitness in pediatric settings. Additional studies with larger samples from more diverse settings and a wider age range are needed to explore associations between longitudinal changes in measures associated with weight status (e.g., abdominal adiposity and cardiometabolic biomarkers) and 90^o^ push-up test performance in youth.

## Data Availability

The datasets supporting the conclusions of this article are available in the Cardiorespiratory and Musculoskeletal Fitness repository, DOI: 10.7303/syn18485159; https://www.synapse.org/#!Synapse:syn18485159/files/.

## Abbreviations

BMI: Body Mass Index
CDC: Centers for Disease Control and Prevention
PACER: Progressive Aerobic Cardiovascular Endurance Run
US: United States
